# A possible role for neutrophils in allergic rhinitis revealed after cellular subclassification

**DOI:** 10.1038/srep43568

**Published:** 2017-03-08

**Authors:** Julia Arebro, Sandra Ekstedt, Eric Hjalmarsson, Ola Winqvist, Susanna Kumlien Georén, Lars-Olaf Cardell

**Affiliations:** 1 Division of ENT Diseases, Department of Clinical Sciences, Intervention and Technology, Karolinska Institutet, Stockholm, Sweden; 2 Department of ENT Diseases, Karolinska University Hospital, Stockholm, Sweden; 3 Department of Medicine, Unit of Translational Immunology, Karolinska Institutet, Stockholm, Sweden

## Abstract

A re-examination of former concepts is required to meet today’s medical challenges in allergic rhinitis. Previously, neutrophils have been treated as a relatively homogenous cell population found in the nose both when the patient is suffering at the height of the allergic season as well as when the patient report no symptoms. However, new data indicates that neutrophils can be divided into different subsets with diverse roles in inflammation. We showed increased levels of neutrophils in peripheral blood, nasal biopsies and nasal lavage fluid (NAL) from allergic patients during the pollen season compared to healthy controls. A closer examination revealed that the activated subset of neutrophils, CD16^high^ CD62L^dim^, outweighed the normal form CD16^high^ CD62L^high^ in nasal tissue among these patients. This skewed distribution was not seen in controls. The normal subset prevailed in peripheral blood from patients as well as controls, whereas CD16^high^ CD62L^dim^ and CD16^dim^ CD62L^dim^ subsets, the latter considered “end state” neutrophils before apoptosis, were elevated in NAL. Functional *in vitro* experiments revealed that activated neutrophils exhibit a T cell priming capacity and an ability to enhance eosinophil migration. Activated neutrophils may thus contribute to allergic inflammation seen in allergic rhinitis by priming T cells and attracting eosinophils.

Allergic rhinitis (AR) is an inflammatory disease of the nasal mucosa, induced by a reaction to a normally harmless antigen. It is characterized by troublesome local nasal symptoms as well as general fatigue, negatively impacting quality of life. In addition, the significant prevalence of AR is associated with staggering indirect costs for the Western societies^1^,^2^,^3^. In order to develop novel strategies for further treatment, traditional concepts of allergic inflammation have to be re-examined^4^.

Eosinophils are well described markers of the allergic reaction, known to play an important role in Th2-mediated immune responses^5^,^6^,^7^,^8^,^9^. Neutrophils have evoked less interest, as they are found in the nose both when the patient is suffering at the height of the allergy season as well as when no symptoms can be reported. Nevertheless the number of neutrophils increases in the nose in symptomatic individuals during the allergy season and their large absolute cell number in comparison with corresponding eosinophils deserves attention^10^,^11^,^12^.

Neutrophils have long been considered short-lived, terminally differentiated cells with a well-established role in acute bacterial infections^13^,^14^. However, information has recently emerged indicating that neutrophils can be divided into different subsets and that the various subsets might have diverse roles in inflammatory diseases. The new subsets are defined by differences in expression of FcγRIII (CD16) and L-selectin (CD62L). Three different subsets have recently been identified: CD16^dim^ CD62L^high^ (immature), CD16^high^ CD62L^high^ (mature) and CD16^high^ CD62L^dim^ (activated and CD11b^bright^ as well as CD11c^bright^)^15^. The same authors reported that the activated subset of mature human neutrophils causes certain immunological responses. Differentiated, CD11b^+^ neutrophils have further been suggested to be reduced at the site of *C. perfringens* infections through systemic reduction of mature neutrophils, possibly explaining polymicrobial infections seen in these patients^16^.

We now suggest that some of these neutrophil subsets might affect the course of inflammation seen in AR. Hence, the present study is designed to identify these subsets as well as determine their ability to traffic between different compartments i.e. peripheral blood, nasal mucosa and nasal lavage fluid (NAL) during the allergic season. Further, the relation between activated neutrophils and CD4^+^ T cells, as well as eosinophils are explored *in vitro* under allergic inflammatory conditions^17^,^18^,^19^,^20^,^21^,^22^.

## Results

### Neutrophils increase in allergy

**Figure 1:**
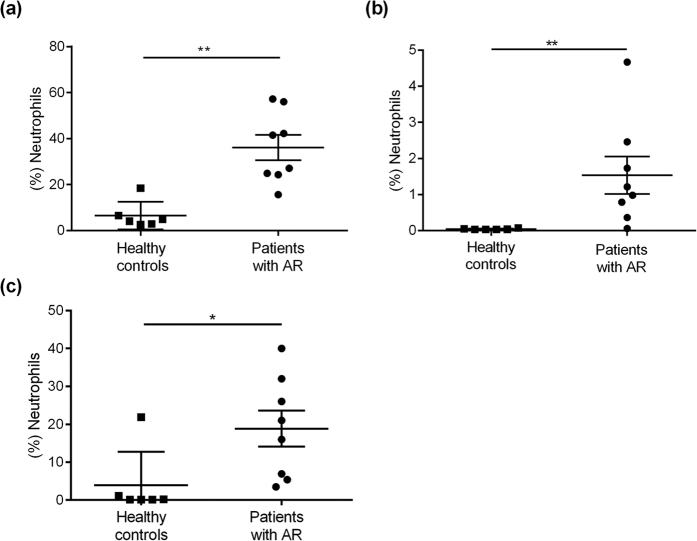
Neutrophil levels. (**a**) Fraction of neutrophils in blood, (**b**) nasal biopsies and (**c**) NAL from patients with AR during pollen season (*n* = 8) and healthy controls (*n* = 6). *Indicates statistical significance (p < 0.05), **indicates statistical significance (p < 0.01) (unpaired t-tests (Mann-Whitney)). Data are shown as mean ± S.E.M.

The first set of experiments investigated the anticipated change in neutrophil number in peripheral blood, nasal biopsies and NAL caused by the pollen season. Neutrophil numbers were found to be enhanced in peripheral blood from patients with AR compared to healthy controls (p < 0.0013) (Fig. 1(a)). Correspondingly, the neutrophil fraction was also significantly increased in nasal biopsies (p < 0.0013) and NAL (p < 0.0127) from patients with AR (Fig. 1(b,c)).

### Different subsets increase in different compartments

**Figure 2:**
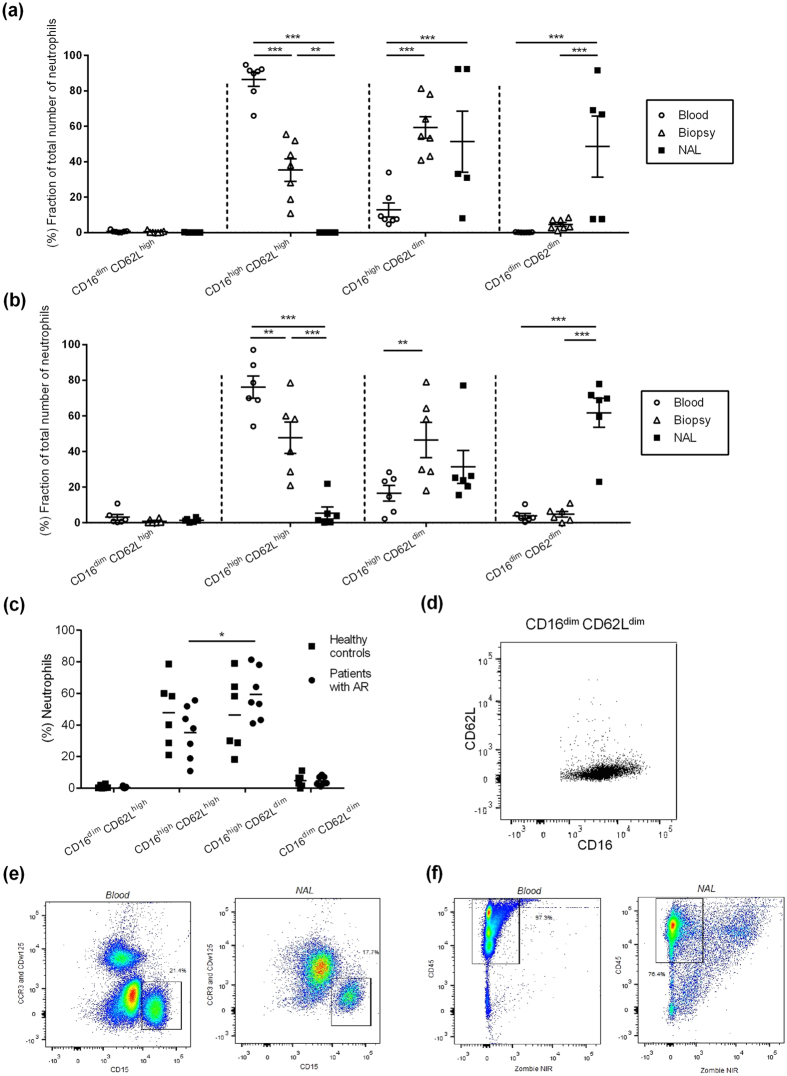
Fraction of different neutrophil subset in different compartments and crucial gating procedures. (**a**) The distribution of neutrophil subsets in blood (*n* = 7), nasal biopsies (*n* = 7) and NAL (*n* = 5) in AR-patients during pollen season and (**b**) in healthy controls (*n* = 6). (**c**) Subset distribution in nasal biopsies. (**d**) NAL incubated with Abs against CD16 and CD62L demonstrating the subset CD16^dim^ CD62L^dim^. Gating procedures of blood and NAL incubated with (**e**) eosinophilic Abs and (**f**) viability experiments. *Indicates statistical significance (p < 0.05), **indicates statistical significance (p < 0.01), ***indicates statistical significance (p < 0.001) (two-way ANOVA with Bonferroni post-test). Data are shown as (a-b) mean ± S.E.M. or (**c**) mean.

In order to further explore the increase in neutrophils in AR patients, the cells were categorised by their expression of CD16 and CD62L (Fig. 2(a)). The phenotypically mature neutrophil subset CD16^high^ CD62L^high^ dominated in peripheral blood. In nasal biopsies, both the mature neutrophil subset, CD16^high^ CD62L^high^, and the activated form CD16^high^ CD62L^dim^, with a hypersegmented nuclear morphology, were elevated (Fig. 2(a)). Finally, the subsets CD16^high^ CD62L^dim^ and CD16^dim^ CD62L^dim^, considered the end state before apoptosis, were elevated in NAL. Healthy controls exhibited the same general compartmental subset pattern as the AR patients with more differentiated subsets in nasal biopsies and even more in NAL compared to in peripheral blood (Fig. 2(b)). It is important to notice that the activated neutrophil subset, CD16^high^ CD62L^dim^, was elevated compared to the mature neutrophil subset CD16^high^ CD62L^high^ in biopsies from patients with AR (p < 0.041) (Fig. 2(c)). This was not seen in healthy controls.

The subset CD16^dim^ CD62L^dim^ has previously not been described (Fig. 2(d)). To ensure that these cells are neutrophils, antibodies were used to exclude eosinophils (Fig. 2(e)). In addition, the majority of cells from peripheral blood and NAL were alive since they were negative for the viability dye Zombie (Fig. 2(f)).

### Activated neutrophils can help activate T cells

**Figure 3:**
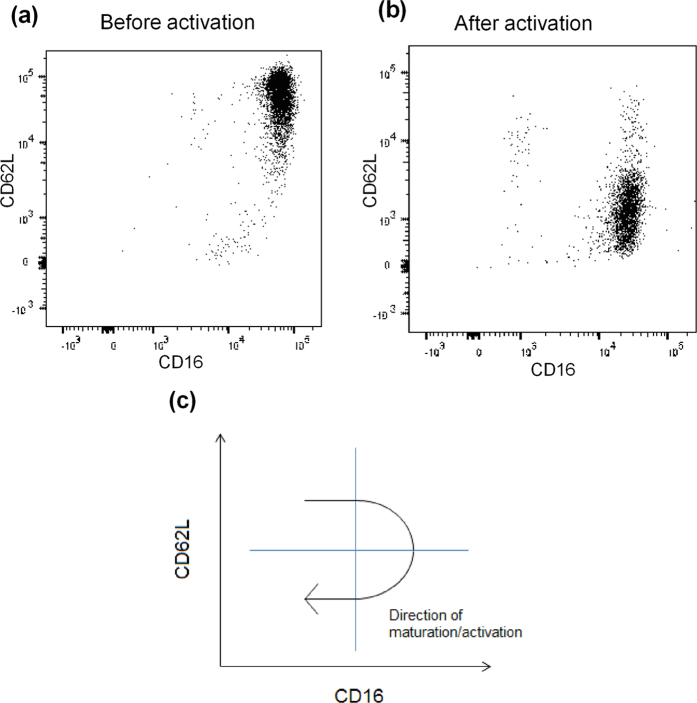
Neutrophils before and after activation. Neutrophils from blood (**a**) before and (**b**) after activation with LPS, TNF-α and IL-8 and incubated with Abs against CD16 and CD62L. (**c**) A schematic figure of how activation affect neutrophils.

**Figure 4:**
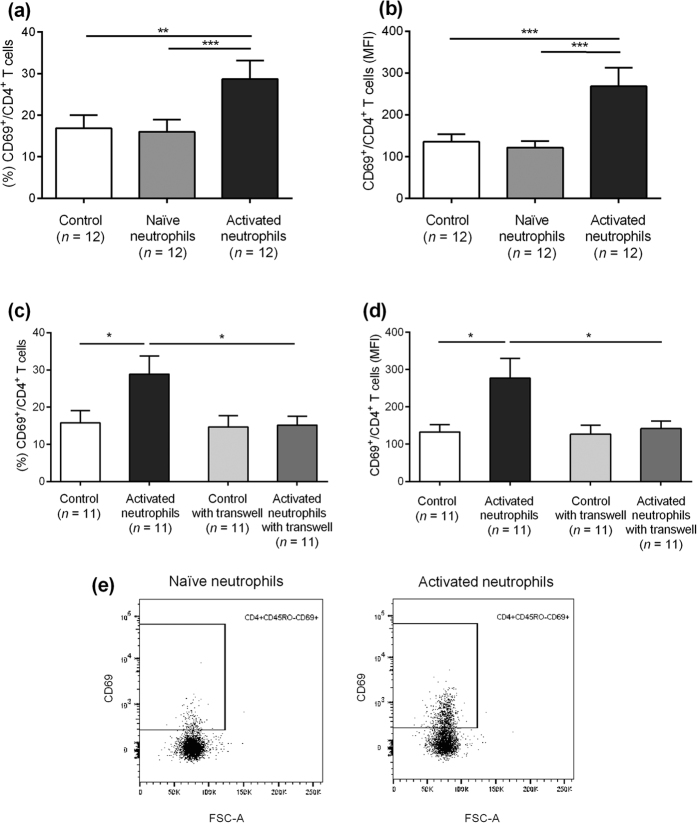
Co-cultures with activated neutrophils and T cells. The (**a**) fraction and (**b**) MFI of CD69^+^/CD4^+^ T cells primed with naïve and activated neutrophils. (**c,d**) Experiments with and without transwell plates. Control = no added neutrophils. (**e**) Representative dot plots illustrating gating procedures. *Indicates statistical significance (p < 0.05), **indicates statistical significance (p < 0.01), ***indicates statistical significance (p < 0.001) (non-parametric (Friedman test) one-way ANOVA with Dunn’s post-test). Data are shown as mean ± S.E.M.

Functional *in vitro* experiments were performed to evaluate the immunological importance of the activated neutrophil subsets. Mature neutrophils, CD16^high^ CD62L^high^ (Fig. 3(a)), were isolated from peripheral blood from healthy controls and activated *in vitro* with LPS, TNF-α and IL-8. Flow cytometric analysis verified an increase of the activated neutrophil subset (CD16^high^ CD62L^dim^) after activation (Fig. 3(b)). The expression of the activation marker CD66b increased simultaneously on neutrophils, supporting their state of activation (see Supplementary Fig. S1). A schematic figure illustrates the phenotypic maturation of neutrophils in the activation process (Fig. 3(c)). After thoroughly washing the neutrophils, isolated autologous T cells from peripheral blood were added in a co-culture. There was an increase in both the fraction of CD69^+^/CD4^+^ T cells (p < 0.0007) (Fig. 4(a)) and in the expression levels of CD69 on CD4^+^ T cells (p < 0.0051) (Fig. 4(b)) when T cells had been primed with activated neutrophils prior to CD3 stimulation. The same findings were made when using blood from patients with AR outside the pollen season (see Supplementary Fig. S2(a,b)). The increased activation of CD4^+^ T cells was confined to CD45RO^−^ T cells indicating that neutrophils specifically affected naïve CD4^+^ T cells (see Supplementary Fig. S3). The T cell activation level in control experiments with naïve neutrophils was comparable with controls using only T cells and CD3, ruling out a T cell priming capacity for naïve neutrophils. Experiments with transwell cell cultures were set up to determine how T cell priming was mediated by activated neutrophils. When using transwell plates, no increase in CD69^+^/CD4^+^ T cells was seen upon priming with activated neutrophils (Fig. 4(c,d)) indicating that the enhanced T cell activation of neutrophils was cell-cell contact or close contact dependent. As a control, monocytes were analysed and no increased activation was seen, making it unlikely that the facilitated T cell activation was mediated by impurities from activated monocytes (see Supplementary Fig. S4). Representative dot plots illustrates the gating procedures (Fig. 4(e)).

### Activated neutrophils mediate eosinophil migration

**Figure 5:**
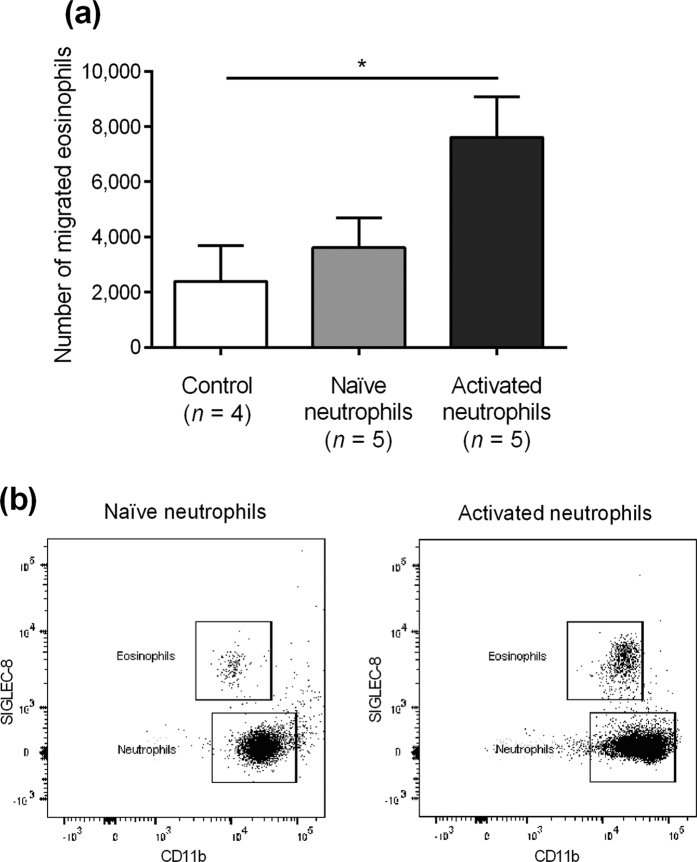
*In vitro* transwell systems with activated neutrophils and eosinophils. (**a**) Eosinophil migration with naïve and activated neutrophils. Control = no added neutrophils. (**b**) Representative dot plots illustrating gating procedures. *Indicates statistical significance (p < 0.05) (one-way ANOVA with Tukey’s post-test). Data are shown as mean ± S.E.M.

To further analyse the importance of neutrophils in allergic inflammation, experiments were set up to study the impact of activated neutrophils on eosinophils. Isolated neutrophils activated *in vitro* with LPS, TNF-α and IL-8 upregulated eosinophilic migration after 3 h of incubation in the transwell system (Fig. 5(a)). Naïve neutrophils had no impact on eosinophil migration. Thus, we concluded that activated neutrophils can increase eosinophil migration, potentially accounting for the local eosinophil infiltration seen in allergy. Representative dot plots illustrates the gating procedures (Fig. 5(b)).

## Discussion

Patients with AR exhibited increased numbers of neutrophils in peripheral blood, nasal biopsies and NAL during the pollen season. When studying this amplification on a subset-level, it was evident that the mature neutrophil subset CD16^high^ CD62L^high^ dominated in peripheral blood while the subsets CD16^high^ CD62L^high^ together with the activated form CD16^high^ CD62L^dim^ were elevated in nasal biopsies. The activated subset CD16^high^ CD62L^dim^ fraction was significantly higher than the normal fraction in nasal tissue from AR-patients. This was not convincingly seen in the healthy controls. Finally, the subsets CD16^high^ CD62L^dim^ and CD16^dim^ CD62L^dim^, considered the end state before apoptosis, were elevated in NAL. A T cell priming capacity of activated neutrophils was demonstrated *in vitro* by co-culturing activated neutrophils and CD4^+^ T cells. Since no such priming was seen using a transwell system, this interaction was likely mediated by close or direct cell-cell contact. Further, activated neutrophils were found to upregulate eosinophil migration.

Special attention has lately been paid to the findings that neutrophil subsets can be defined by differences in CD16 and CD62L expression^15^,^23^. The CD16^high^ CD62L^dim^ neutrophil subset has previously been suggested to cause T cell inhibition through Mac-1 resulting in immunosuppression^15^. However, the way in which different neutrophil subsets influence various diseases has scarcely been investigated. We have previously demonstrated that activated neutrophils seem to have anti-tumorigenic properties^24^. Moreover, we have suggested that the enhanced systemic adaptive immune response seen among patients with AR might protect against head and neck squamous cell carcinoma, a phenomenon likely mediated by peripheral blood mononuclear cells^25^. The same subtype has been demonstrated in other cancer forms^26^. Neutrophils from allergic patients have been shown to downregulate surface expression of CD62L upon allergen stimulation^27^. The distribution of different neutrophils based on their expression of CD16 and CD62L has to our knowledge however never been studied in allergy.

Both neutrophils and eosinophils are known to increase upon allergen exposure. Even though the neutrophils far outnumber the amount of eosinophils found both locally and systemically during symptomatic AR, the former has been given much less attention as potential players in the allergic reaction (Fig. 1). This is probably due to the fact that neutrophils can be found in the nose in symptomatic patients at the height of the allergic season as well as in the asymptomatic phase. However, when examining changes in neutrophil subsets in different compartments, a different picture emerges (Fig. 2). It becomes clear that activated and differentiated neutrophil subsets accumulate at the site of allergic provocation, in this case in the nose. Furthermore, we demonstrated that these activated neutrophils have the ability to prime CD4^+^ T cells, a phenomenon known to be of great importance for the start of allergic inflammation at the site of antigen exposure^28^,^29^. This notion is supported by a recent publication demonstrating that timothy grass pollen can stimulate neutrophil immune responses through the secretion of IL-8^11^. In addition, the T cells in our experiment were CD45RO^−^ negative, indicating that the activated neutrophil subset could have a role in the allergic sensitisation process, by affecting naïve antigen-specific CD4^+^ T cells^30^. Hence, locally activated neutrophils in the upper airways seem to have the ability to mediate the inflammatory process in allergy. Further, neutrophils have recently been shown to be components of the response to, immunisation accumulating in lymph nodes and forming an adaptive immune response^31^. Neutrophil subsets could even partly explain the airway epithelial injury seen in asthma^32^,^33^. Altogether, these reports suggest that neutrophils are more complex than previously thought.

T cell activation and proliferation is central in allergic immunity. However, it is still not completely understood which cells are responsible for local mucosal T cell activation. It has previously been shown that it takes several days to recruit conventional dendritic cells to the nasal mucosa upon allergen challenge^34^. We have earlier shown the immunological importance of nasal epithelial cells activating T cell at the site of allergen exposure, i.e. the nose^35^. In addition, previous publications in mice have showed that neutrophils can influence CD8^+^ T cell response and possibly also direct or prime Th1 T cell responses^36^,^37^,^38^. The current experiments demonstrate that activated human neutrophils can prime T cells, thus facilitating CD3 activation. No such priming was seen when naïve neutrophils were used or when T cells and CD3 were used alone speaking against potential contamination from monocytes. Separate analyses of monocytes in wells containing activated neutrophils demonstrated no monocyte activation, definitively ruling out a role for monocytes in facilitating T cell activation (see Supplementary Fig. S4). These assays identify that activated neutrophils have stimulatory properties on T cells, further suggesting the possible functional impact of locally activated neutrophil subsets in allergic patients.

The presence of both circulating and local eosinophils during ongoing allergic inflammation is well established^39^. In contrast, the detailing of their pharmacological suppression and the resulting clinical improvement has over the years been limited^40^,^41^. Interestingly, a significant decrease of not only eosinophils but also neutrophils in the nasal mucosa was seen upon sublingual immunotherapy (IT) to *Parietaria* species^42^. Neutrophils were also shown to be decreased in a skin chamber model upon AR IT^43^,^44^. Further, IL-9 positive neutrophils increased in the nasal mucosa during pollen season, something that could be successfully inhibited by IT^45^.

The present data on neutrophil subsets might be a next step in understanding the great infiltration of neutrophils in the nasal mucosa in allergy. We demonstrate the ability for activated subsets to directly prime T cells and enhance migration of eosinophils^46^,^47^. This is in line with previous publications suggesting a more complex role for neutrophils^28^,^29^,^31^,^46^. Further experimental studies depleting or deactivating the activated neutrophils in an allergic setting, potentially affecting the course of the inflammation, are required. This could open up new therapeutic possibilities for AR.

## Methods

### Patients

Eight non-smoking AR patients with allergy to birch pollen (*n* = 3), grass pollen (*n* = 1) or both (*n* = 4) were included in the study (mean age 28 years; M:F 2:6). All patients were healthy with the exception of their allergy. Their allergy was diagnosed on the basis of clinical history and positive skin prick test (SPT) and/or the radioallergosorbent test (RAST) for allergen-specific IgE. Samples were taken in-season and all patients had nasal symptoms of allergy at the time of participation. Seven patients had been off antihistamine treatment for more than 48 h, one had been off antihistamine treatment for more than 24 h. Four patients reported no use of topical nasal steroids for at least a month. Four patients had been off topical nasal steroids for more than three days. No patient had been on inhaled steroids for at least one month. In addition, six healthy non-smoking controls were included (mean age 21 years; M:F 2:4). RAST for allergen-specific IgE were negative for all healthy controls included in the study. The patients and healthy controls reported no history of infections in the airways or in the rest of the body three weeks prior to participating. The study was approved by the local ethical committee in Stockholm, Karolinska Institutet, and all patients and healthy controls gave their written informed consent. All methods were performed in accordance with the relevant guidelines and regulations.

### Human nasal biopsies

Nasal biopsies were taken from inferior turbinates of healthy non-smoking controls under local anaesthesia as previously described^48^. Tissue was put through a 100 μm cell strainer (BD Falcon, Franklin Lakes, NJ, USA), into Dulbecco’s Modified Eagle’s Medium and Nutrient Mixture Ham’s F12 Medium (DMEM/F-12) (Gibco^®^, Paisley, UK) containing 10% FBS and incubated at RT for 5 min. Cells were washed and centrifuged and the pellet was resuspended in PBS containing 2% FBS prior to analysis with flow cytometry.

### Human peripheral blood

Peripheral blood was collected in heparin tubes, lysed with formic acid and ion solution, and finally stained with different antibodies for flow cytometry (see Antibodies and flow cytometry section for details).

### Recovery of nasal lavage fluid

Nasal lavage fluid was collected as previously described^48^. In brief, after cleaning excess mucous by forceful exsufflation, 8–10 ml of sterile saline solution (0.9% NaCl) at RT was aerosolised into the nostrils and passively collected from the nostrils. When 7 ml of fluid were recovered into a graded test tube, it was centrifuged for 10 min. The pellet was resuspended in PBS containing 2% FBS before analysis with flow cytometry.

### T cell stimulation

For all *in vitro* experiments, healthy controls with no known allergies were recruited. For the T cell stimulation experiments, patients with birch and/or grass pollen allergy were also recruited. All participants were non-smokers and all patients were healthy with the exception of their allergy. Blood was collected in heparin tubes. T cells and neutrophils were isolated from peripheral blood using ficoll-paque (Sigma-Aldrich, St. Louis, MO, USA) according to the manufacturer’s instructions. The erythrocytes in the granulocyte rich pellet were lysed (0.8% NH_4_Cl, 10 mM KHCO_3_ and 0.1 mM EDTA). Neutrophils were further purified with CD15 microbeads (Miltenyi Biotec, Bergisch Gladbach, Germany) according to the manufacturer’s instructions. The neutrophils were diluted to a concentration of 2 × 10^6 ^cells/ml in TexMACS Medium (Miltenyi Biotec) containing 10% autologous plasma, 100 U/mL penicillin and 100 μg/mL streptomycin (Life Technologies, Eugene, OR, USA). Thereafter, the neutrophils were activated with 1 μg/ml LPS (product no: L2654, Sigma-Aldrich, St. Louis, MO, USA), 5 ng/ml TNF-α and 10 ng/ml IL-8 (R&D System, Minneapolis, MN, USA) for 15 min and then washed with PBS twice. The lymphocyte interface from the ficoll-paque isolation step was collected and washed with PBS. The cells were diluted to a concentration of 2 × 10^6 ^cells/ml in the same medium as the neutrophils.

A co-culture with activated neutrophils (5 × 10^5^) and lymphocytes (5 × 10^5^) was set up in wells for 30 min. Thereafter, CD3 (0.05 μg) (clone OKT3, BioLegend^®^, San Diego, CA, USA) was added and incubated for 90 min, for T cell activation. The cell suspension was then collected and analysed with flow cytometry.

For blocking cell-cell contact, transwell plates (0.4 μm, Corning, NY, USA) were used in separate experiments to separate neutrophils from lymphocytes. In these experiments, CD3 was added to the lymphocyte part of the transwell plates.

Control experiments with naïve neutrophils were run in all experiments.

### Eosinophil migration experiment

Eosinophils and neutrophils were collected from whole blood using MACSxpress Neutrophil Isolation Kit, human and Eosinophil Isolation Kit, human, (Miltenyi Biotec) according to the manufacturer’s instructions. Remaining erythrocytes were lysed. Cells were diluted in Complete Medium (CM) consisting of RPMI 1640 (Invivogen, San Diego, CA, USA) with 10% autologous plasma, penicillin 100 U/mL and streptomycin 100 μg/mL (Invitrogen, Carlsband, CA, USA), to a concentration of 1 × 10^5 ^cells/ml. Migration set up was conducted with transwell plates (3.0 μm PTFE Collagen Coated Membrane, Corning, NY, USA). Cells were added (5 × 10^5^ neutrophils in the bottom well and 2.5 × 10^5^ eosinophils in the insert) and incubated for 3 h at 37 °C in a humidified 5% CO_2_ air atmosphere. Inserts were removed and cell suspension in the wells were analysed with flow cytometry using CountBright TM Absolute Counting Beads (Life Technologies, Eugene, OR, USA).

### Antibodies and flow cytometry

The following monoclonal antibodies were purchased from BD Biosciences (San Jose, CA, USA): anti-human CD4 (clone RPA-T4), CD8 (clone RPA-T8), CD5 (clone UCHT2), CD11b (clone ICRF44), CD14 (clone M5E2), CD15 (clone W6D3), CD16 (clone 3G8), CD45 (clone 2D1), CD45RA (clone HI100), CD45RO (clone UCHL1), CD56 (clone B159), CD62L (clone DREG-56), CD69 (clone FN50), CD80 (clone L307,4 and BB1), CD86 (clone 2331), CCR3 (clone 5E8) and CDw125 (clone A14). Anti-human HLA-DR (clone L243), CD43 (clone MEM-59) and SIGLEC-8 (clone 7C9) were purchased from BioLegend^®^ (San Diego, CA, USA). Anti-human ICAM-1 (clone BBIG-I1) was purchased from R&D (Minneapolis, MN, USA) and Zombie NIR™ Fixable Viability Kit was purchased from BioLegend^®^. All antibodies were titrated for optimum concentration before use, with dilutions ranging from 1:5 to 1:400. Cells were identified based on forward and side scatter properties. In flow cytometry assays studying CD69, fluorescence minus one (FMO) controls were used. Cells were analysed on an LRSFortessa analyser (BD Biosciences) and data were processed using FlowJo software (©Tree Star, Inc., Ashland, USA).

### Statistics

Statistical differences between patients with allergy and healthy controls were performed using unpaired t-tests (Mann-Whitney). For more than two sets of matched data, a two-way ANOVA with Bonferroni post-tests was performed. For the *in vitro* experiments, a non-parametric (Friedman test) one-way ANOVA was used together with Dunn’s post-test, except for the eosinophil migration experiments where Tukey’s multiple comparisons test was used. A p-value of 0.05 or less was considered statistically significant (*p < 0.05, **p < 0.01, ***p < 0.001). Statistical analyses were performed using GraphPad Prism software (version 6.0, GraphPad Software, La Jolla, CA). All data are shown as mean ± S.E.M. For human data, *n* equals the number of patients.

## Additional Information

**How to cite this article**: Arebro, J. *et al*. A possible role for neutrophils in allergic rhinitis revealed after cellular subclassification. *Sci. Rep.* **7**, 43568; doi: 10.1038/srep43568 (2017).

**Publisher's note:** Springer Nature remains neutral with regard to jurisdictional claims in published maps and institutional affiliations.

## Supplementary Material

Supplementary File
