# Supplementary material for: A possible role for neutrophils in allergic rhinitis revealed after cellular subclassification
Source: Sci Rep. 2017 Mar 8;7:43568. doi: 10.1038/srep43568 (PMC5341103; doi:10.1038/srep43568)

A possible role for neutrophils in allergic rhinitis revealed after cellular subclassification

Julia Arebro, Sandra Ekstedt, Eric Hjalmarsson, Ola Winqvist, Susanna Kumlien Georén, Lars-Olaf Cardell

SUPPLEMENTARY FILE

SUPPLEMENTARY FIGURE LEGENDS

Fig. S1. Flow cytometry data. The expression of the activation marker CD66b correlated to activation level of neutrophils.

Fig. S2. Co-cultures with activated neutrophils and T cells of AR-patients. The (**a**) fraction and (**b**) MFI of $CD69^{+}/CD4^{+}$ T cells primed with naïve and activated neutrophils. Control = no added neutrophils. *Indicates statistical significance ($p < 0.05$) (non-parametric (Friedman test) one-way ANOVA with Dunn's post-test). Data are shown as mean ± S.E.M.

Fig. S3. Gating procedures. Gating procedures of T cells, verifying the $CD4^{+}$ T cells to be highly $CD45RO^{-}$.

Fig. S4. Monocyte analysis. Analysis of monocyte impurities of T cell experiments revealing no increased activation in the wells containing activated neutrophils compared to control wells without neutrophils or with naïve neutrophils. Staining with Abs against CD14, CD80, CD86 and HLA-DR.

Supplementary Figure S1

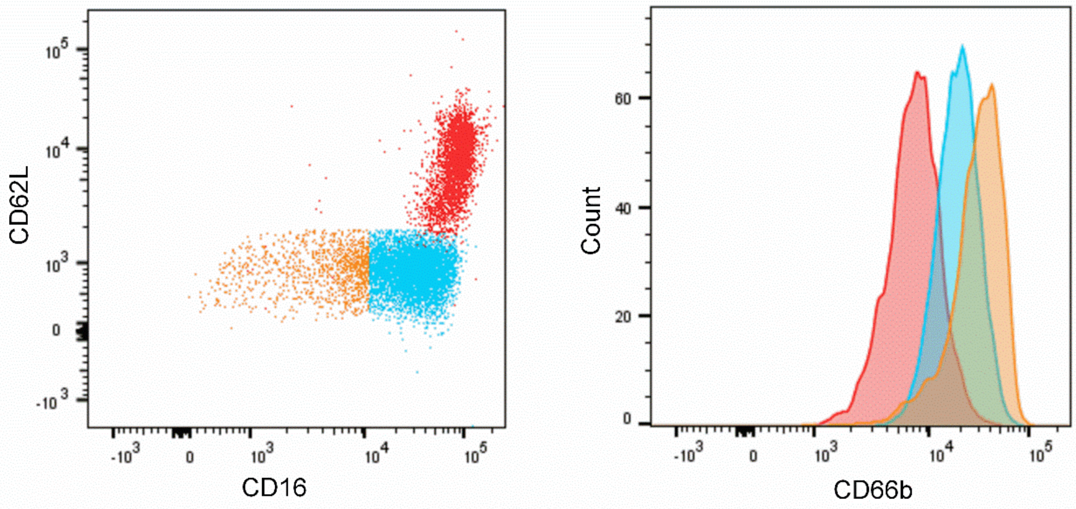

Neutrophil subset	MFI CD66B
CD16^{high} CD62L^{high} (red)	8,046
CD16^{high} CD62L^{dim} (blue)	20,503
CD16^{dim} CD62L^{dim} (orange)	32,292

Supplementary Figure S2

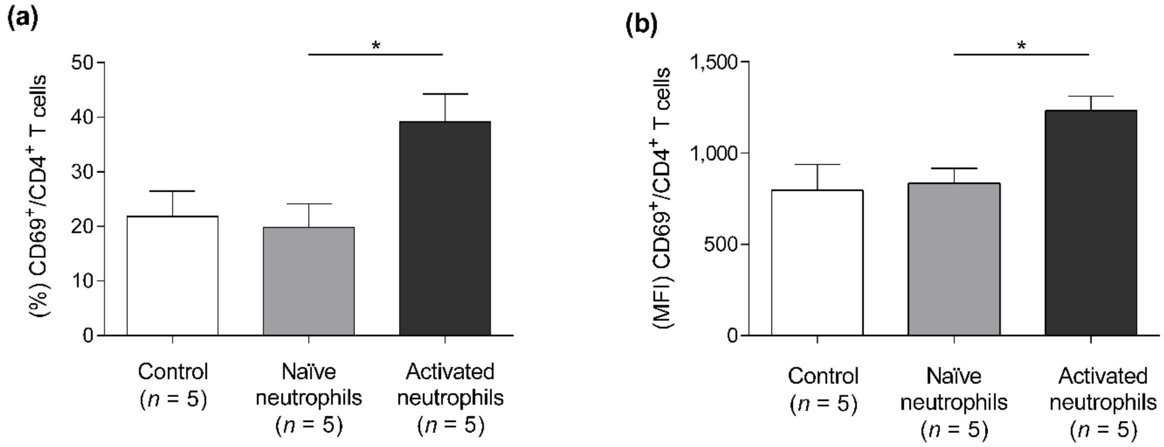

Supplementary Figure S3

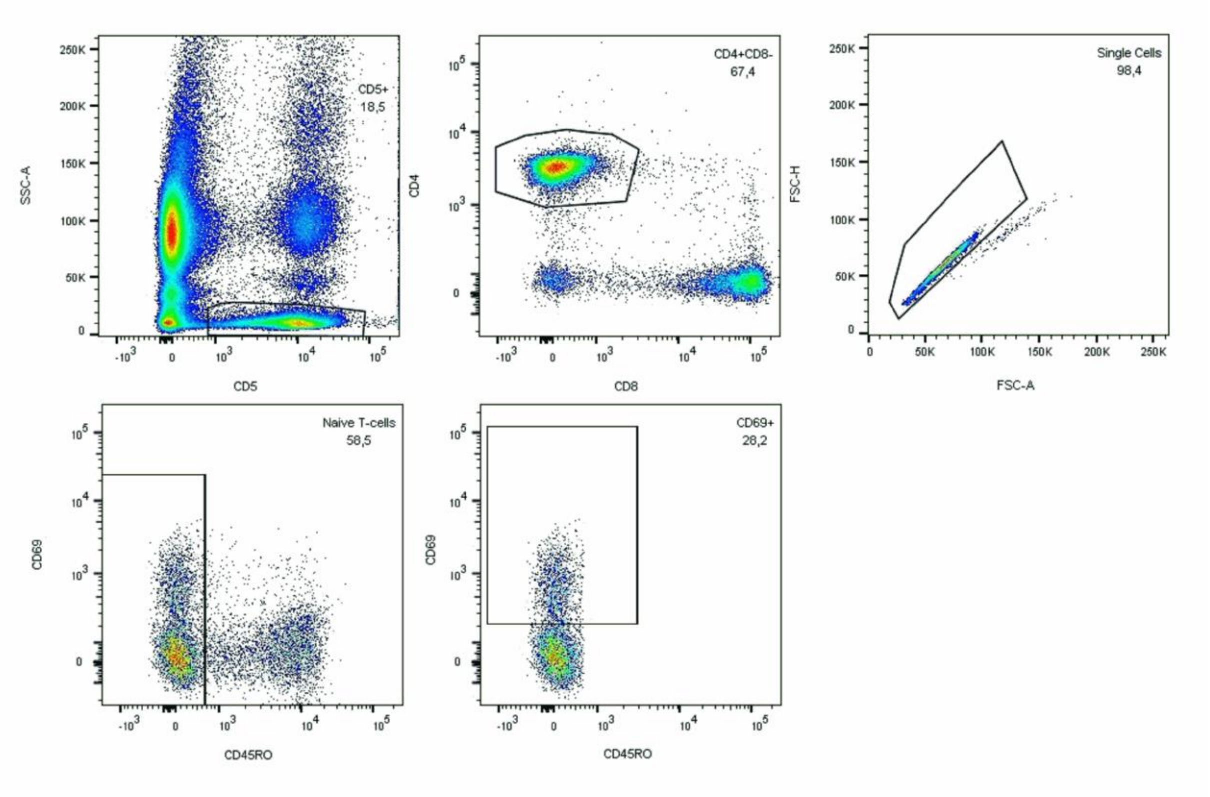

Supplementary Figure S4

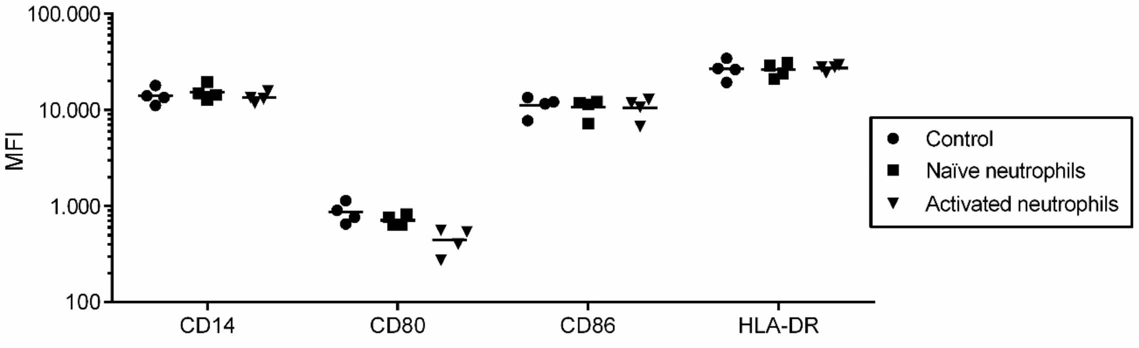

Supplement: Supplementary File [file srep43568-s1.pdf]
